# A Case of Recurrent Gastric Cancer in Which Rheumatoid Arthritis and Sjögren's Syndrome Became Evident With Nivolumab

**DOI:** 10.1002/ccr3.73855

**Published:** 2026-11-03

**Authors:** Haruhiko Takemoto, Keiji Nishibeppu, Takeshi Kubota, Hiroyuki Inoue, Kazuya Takabatake, Hirotaka Konishi, Hitoshi Fujiwara, Atsushi Shiozaki

**Affiliations:** ^1^ Division of Digestive Surgery, Department of Surgery Kyoto Prefectural University of Medicine Kyoto Japan; ^2^ Iseikai International General Hospital Osaka Japan

**Keywords:** gastric cancer, immune‐related adverse events, nivolumab, rheumatoid arthritis, Sjögren's syndrome

## Abstract

A 59‐year‐old man with recurrent gastric cancer began nivolumab as third‐line therapy and subsequently experienced joint pain and dry eyes. Symptoms worsened despite NSAIDs. He was diagnosed with rheumatoid arthritis and Sjögren's syndrome, associated with nivolumab, and was successfully treated with immunosuppressive and anti‐rheumatic medications.

## 1 Introduction

Programmed cell death protein 1 (PD‐1) is a receptor expressed on activated T and B lymphocytes, macrophages, dendritic cells, and monocytes [1]. It plays a key role in maintaining immune tolerance by inhibiting adaptive and innate immune responses [2]. However, this receptor is exploited by carcinoma cells to evade the immune system [3]. PD‐1 inhibitors, including nivolumab, are used in cancer therapy to enhance antitumor immunity by blocking the PD‐1 pathway. This is why PD‐1 inhibitors can make immune response exaggerated and induce immune‐related adverse events (irAEs), including interstitial pneumonia, colitis, myositis, arthritis, and other conditions [4, 5].

The CheckMate 649 trial and ATTRACTION‐4 trial showed that nivolumab plus chemotherapy improved progression free survival significantly in advanced gastric and gastro‐esophageal junction adenocarcinoma patients compared to chemotherapy alone [6, 7]. The role of PD‐1 inhibitors, including Nivolumab, has become more vital in cancer treatment, while more irAEs have been reported.

We report a case of nivolumab‐associated rheumatoid arthritis (RA) and Sjögren's syndrome.

## 2 Case Presentation/Examination

A 59‐year‐old man with no notable medical history was diagnosed with GC and underwent laparoscopic distal gastrectomy, D2 lymph node dissection, and Roux‐en‐Y reconstruction. The stage of GC was T3N2M0 pStage IIIA according to the Union for International Cancer Control (UICC) 8th edition [8].

The patient received eight courses of S‐1 plus oxaliplatin (SOX) as adjuvant chemotherapy over 6 months. Six months after completing the adjuvant chemotherapy, the patient's carcinoembryonic antigen (CEA) levels increased to 28.4 ng/mL, and contrast‐enhanced computed tomography (CT) revealed peritoneal dissemination (Figure 1A,B). Seven courses of SOX were administered as first‐line therapy and the CEA levels decreased to 9.6 ng/mL. However, the CEA levels increased again to 16.1 ng/mL, and the CT showed new peritoneal nodules (Figure 1C). Consequently, second‐line therapy with paclitaxel (PTX) and ramucirumab (RAM) was initiated, leading to the decrease of peritoneal nodules and a decrease in CEA levels to 7.6 ng/mL (Figure 1D). Fluorodeoxyglucose‐positron emission tomography (FDG‐PET/CT) showed no abnormal uptake in the peritoneal lesions, and the treatment was therefore suspended. Approximately 4 months later, CEA levels increased again and CT revealed recurrent peritoneal nodules; PTX plus RAM was therefore resumed and administered for 13 courses. After 13 courses of PTX plus RAM therapy, the CEA levels increased again and a subcutaneous 18.0 mm nodule located under the umbilical port scar appeared (Figure 2A). Then, nivolumab was administered as third‐line therapy (Figure 3).

**FIGURE 1:**
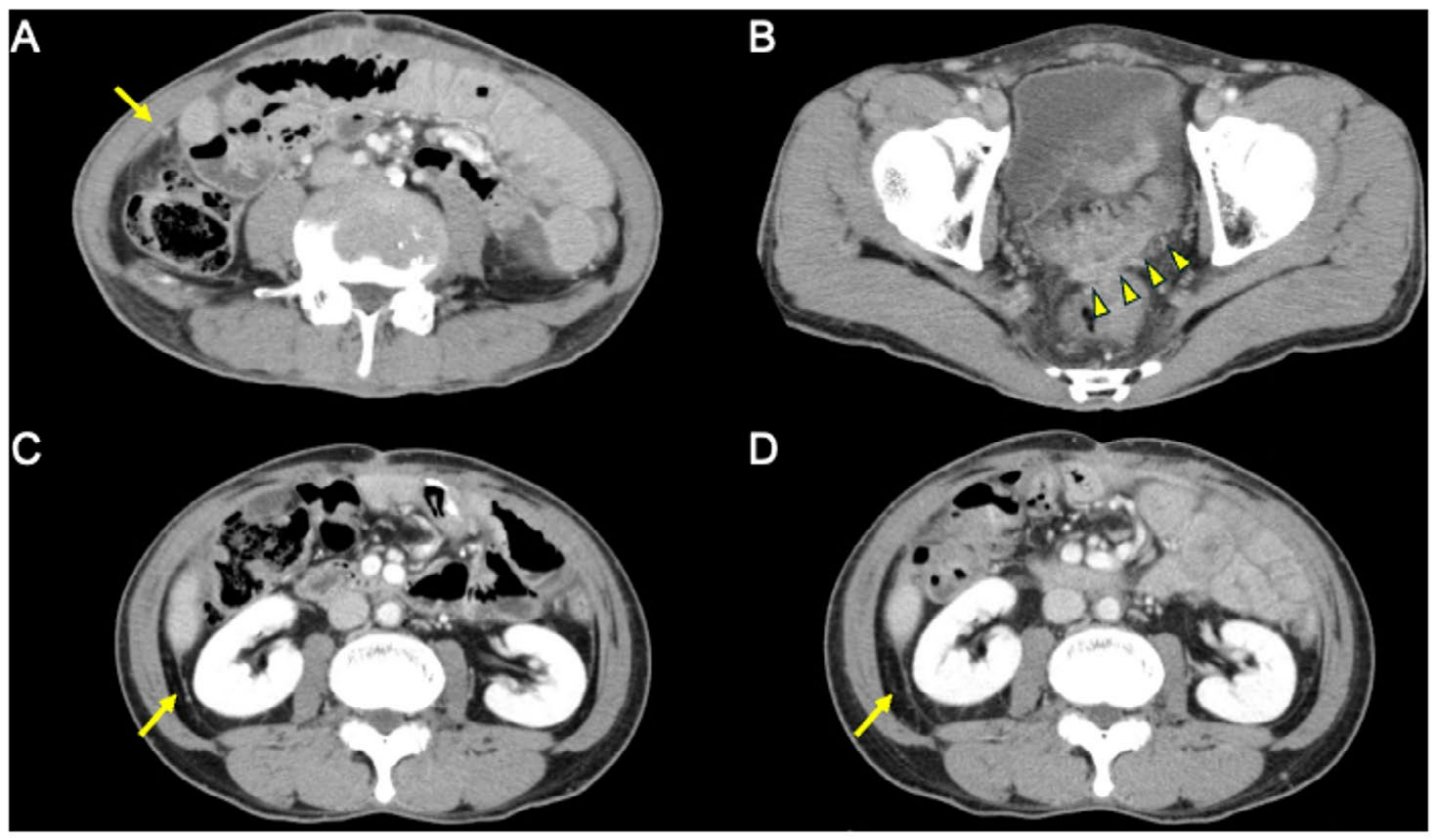
Abdominal CT findings. (A) A peritoneal nodule (arrow). (B) Peritoneal thickening and enhancement (arrowheads). (C) Enhancing peritoneal nodules (arrow). (D) Decrease in the size of the peritoneal nodules (arrow).

**FIGURE 2:**
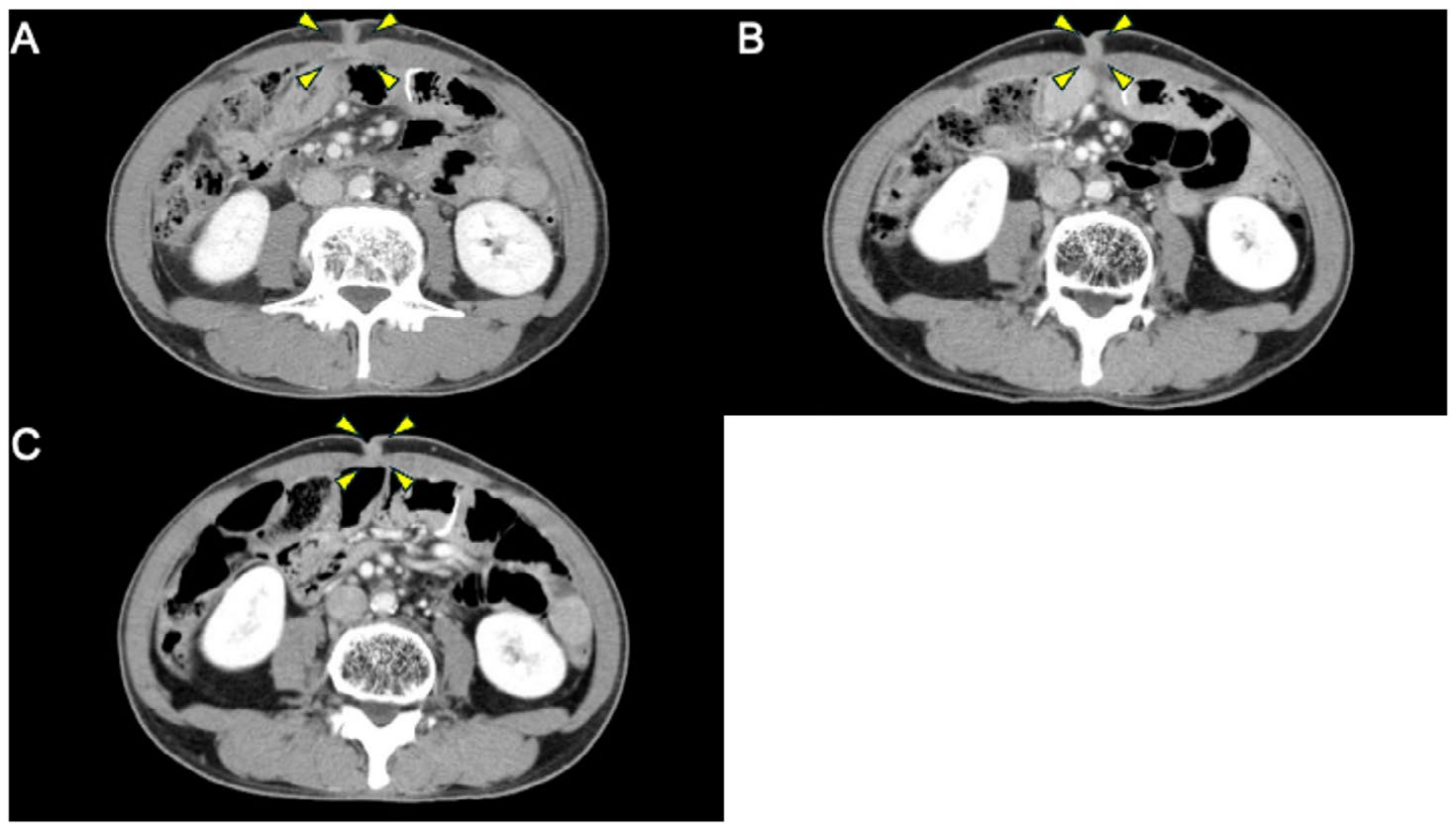
CT findings of the port‐site under the umbilical port scar. (A) A subcutaneous nodule located under the umbilical port scar, and an enhancing nodule between the rectus abdominis muscles (arrowheads). (B) Decrease in the size of the subcutaneous nodule (arrowhead). (C) No change in the size of the subcutaneous nodule (arrowhead).

**FIGURE 3:**
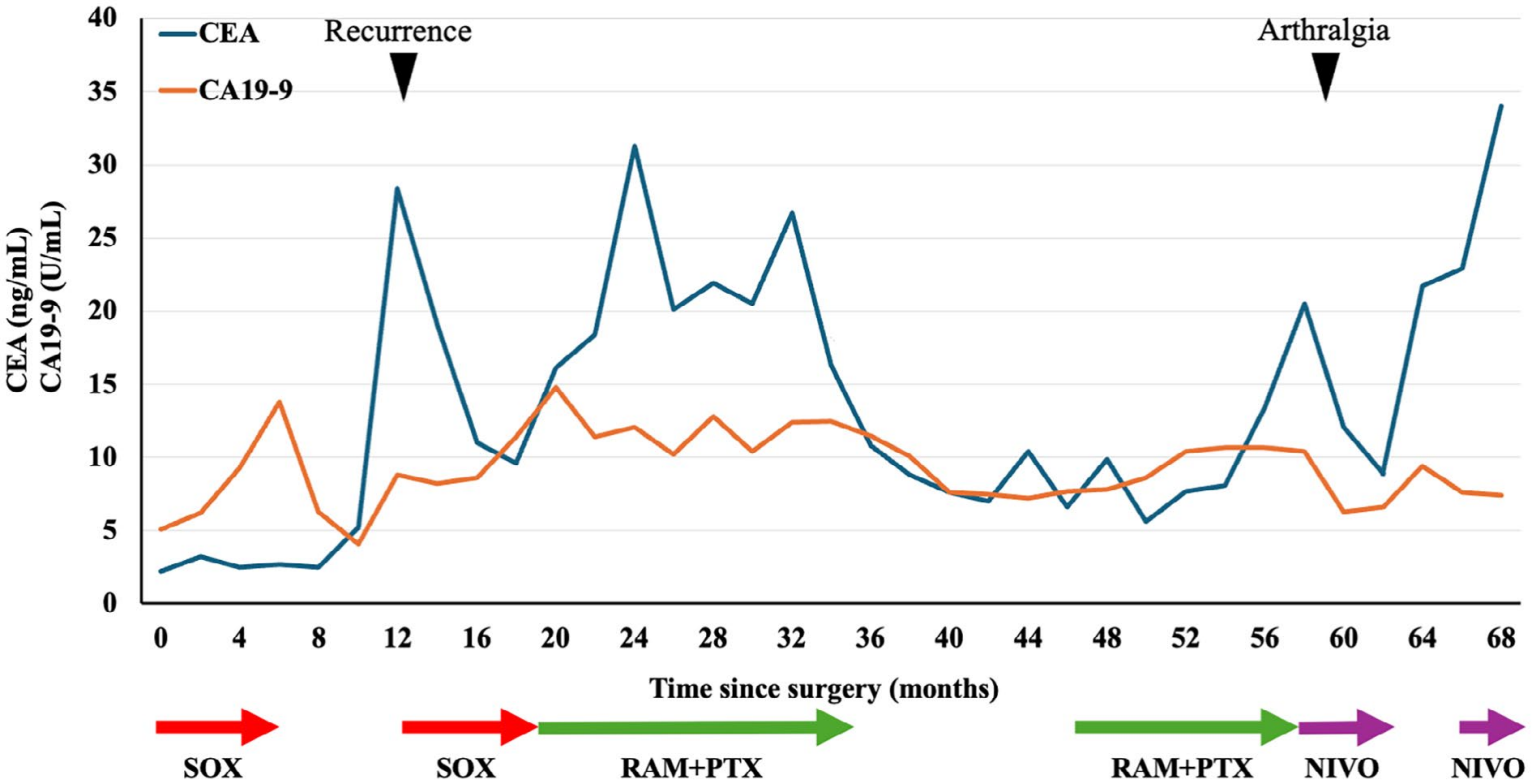
Changes in CEA and CA19‐9 levels after surgery. Six months after completing adjuvant therapy, CEA levels increased to 28.4 ng/mL and peritoneal dissemination was detected. Seven courses of SOX were administered as first‐line therapy and the CEA level decreased to 9.6 ng/mL. CEA levels subsequently increased to 16.1 ng/mL, and PTX plus RAM was initiated as second‐line therapy, leading to a decrease in the peritoneal nodules and in CEA levels to 7.6 ng/mL. Because FDG‐PET/CT showed no abnormal uptake in the peritoneal lesions, the treatment was suspended. When CEA levels increased again and CT revealed new peritoneal nodules, PTX plus RAM was resumed and administered for 13 courses. Thereafter, CEA levels increased again, and nivolumab was administered as third‐line therapy. CA19‐9, Carbohydrate antigen 19‐9; CEA, carcinoembryonic antigen; CT, computed tomography; FDG‐PET/CT, fluorodeoxyglucose‐positron emission tomography/computed tomography; NIVO, nivolumab; PTX, paclitaxel; RAM, ramucirumab; SOX, S‐1 plus oxaliplatin.

Before the initiation of nivolumab, a general interview regarding the history of autoimmune and collagen diseases was performed. The patient had no personal or family history of autoimmune diseases. Since the patient was completely asymptomatic without any rheumatologic complaints, autoimmune serologies, including RF, anti‐CCP antibodies, anti‐SS‐A and anti‐SS‐B antibodies, and antinuclear antibodies, had not been evaluated prior to treatment. After two courses of nivolumab, the patient developed arthralgia in the proximal interphalangeal (PIP) joints, which was considered to be a rheumatic irAE and was assessed as Grade 1 inflammatory arthritis according to the ASCO guideline [9]. Loxoprofen was initiated and nivolumab was continued in accordance with the guideline. Although the joint pain improved temporarily with loxoprofen, the patient developed non‐swelling arthralgia in the left wrist after four courses, followed by knee pain after six courses. These symptoms remained Grade 1, did not limit activities of daily living, and were manageable with NSAIDs; nivolumab was therefore continued in accordance with the ASCO guideline [9]. The nodule under the umbilical port scar had also decreased in size to 11.1 mm, indicating a clinical response to nivolumab (Figure 2B). Following the ninth course of nivolumab, the joint pain worsened, accompanied by the onset of shoulder pain, left wrist swelling, morning stiffness, and dry eyes. Due to these symptoms, the patient was referred to the Department of Inflammation and Immunology.

## 3 Methods

Physical examination revealed swelling and tenderness in the wrists, shoulders, elbows, knees, ankles, proximal interphalangeal joints, and metacarpophalangeal joints.

The differential diagnoses included irAE‐related arthritis and RA. Sicca symptoms due to other causes, such as medications, were also considered.

Blood tests showed anemia (Hb 11.7 g/dL), hypoalbuminemia (Alb 3.5 g/dL), and elevated C‐reactive protein (0.49 mg/dL). Serum markers for autoimmune diseases were positive: RF (406 U/mL; reference value under 15 IU/mL), anti‐cyclic citrullinated peptide (CCP)‐Ab (500 U/mL; reference value under 4.5 U/mL), and anti‐Sjögren's syndrome A (SS‐A)‐Ab (16.3 U/mL; reference value under 7.0 U/mL). The Schirmer test, used to diagnose dry eye, was positive in both eyes (1 mm in the right eye, 3 mm in the left eye; positive, ≤ 5 mm/5 min). The chewing gum test was also positive (10 mL/10 min; positive, ≤ 10 mL/10 min), although this test is not included in the 2016 ACR/EULAR classification criteria. Based on these clinical and laboratory findings, the patient was diagnosed with RA and Sjögren's syndrome according to the 2010 ACR‐EULAR RA Classification Criteria [10] and the 2016 ACR‐EULAR Sjögren's syndrome Classification Criteria [11], respectively (Table 1).

**TABLE 1:** Classification criteria for rheumatoid arthritis and Sjögren's syndrome, and the scores in the present case.

【2010 ACR‐EULAR RA Classification Criteria】	Points	【2016 ACR‐EULAR SS Classification Criteria】	Points
Joint Involvement		Labial salivary gland with focal lymphocytic sialadenitis and focus score ≧ 1	3
1 large joint	0	Anti‐SS‐A/Ro antibody	**3**
2–10 large joint	1	Ocular surface staining score ≥ 5 on at least one eye	1
1–3 small joints	2	Schirmer ≤ 5 mm/5 min on at least one eye	**1**
4–10 small joints	3	Unstimulated whole saliva flow rate ≤ 0.1 mL/min	1
> 10 joints (at least 1 small joint)	**5**		
Serology (need at least 1)			
Negative RF, negative anti CCP‐Ab	0		
Low positive RF or low positive anti CCP‐Ab	2		
High positive RF or high positive anti CCP‐Ab	**3**		
Acute phase reactants (need at least 1)			
Normal CRP and normal ESR	0		
Abnormal CRP or abnormal ESR	**1**		
Duration of symptoms			
< 6 weeks	0		
≧ 6 weeks	**1**		
≧ 6/10: definite RA	**10**	≧4/9: definite SS	4

*Note:* The patient fulfilled the 2010 ACR/EULAR classification criteria for rheumatoid arthritis with a total score of 10 of 10 (joint involvement, 5; serology, 3; acute‐phase reactants, 1; duration of symptoms, 1), and the 2016 ACR/EULAR classification criteria for Sjögren's syndrome with a total score of 4 of 9 (anti‐SS‐A/Ro antibody, 3; Schirmer test, 1). A labial salivary gland biopsy, ocular staining score, and unstimulated whole saliva flow rate were not evaluated. The patient had symptoms of ocular dryness and none of the exclusion criteria of the 2016 criteria. Criteria from references [10, 11]. Bold values indicate the criteria fulfilled by the present patient.

Abbreviations: ACR, American College of Rheumatology; CCP, cyclic citrullinated peptide; CRP, C‐reactive protein; ESR, erythrocyte sedimentation rate; EULAR, European League Against Rheumatism; RA, rheumatoid arthritis; RF, rheumatoid factor; SS, Sjögren's syndrome.

Treatment with prednisolone (15 mg/day) and methotrexate (6 mg/week) was initiated. At the first follow‐up visit after starting methotrexate, chest radiography revealed a very small left pleural effusion. Because pleural effusion is a contraindication to methotrexate owing to the risk of delayed drug elimination and increased toxicity, methotrexate was discontinued after two doses and replaced with iguratimod (25 mg/day). No further investigations, such as thoracentesis or chest CT, were performed because the effusion was very small and the patient had no respiratory symptoms; the effusion resolved spontaneously on follow‐up chest radiography. Prednisolone was tapered to 2.5 mg/day over 13 weeks and subsequently discontinued. Bucillamine (200 mg/day) was added at 13 weeks, and the RA was subsequently controlled with iguratimod and bucillamine (Figure 4).

**FIGURE 4:**
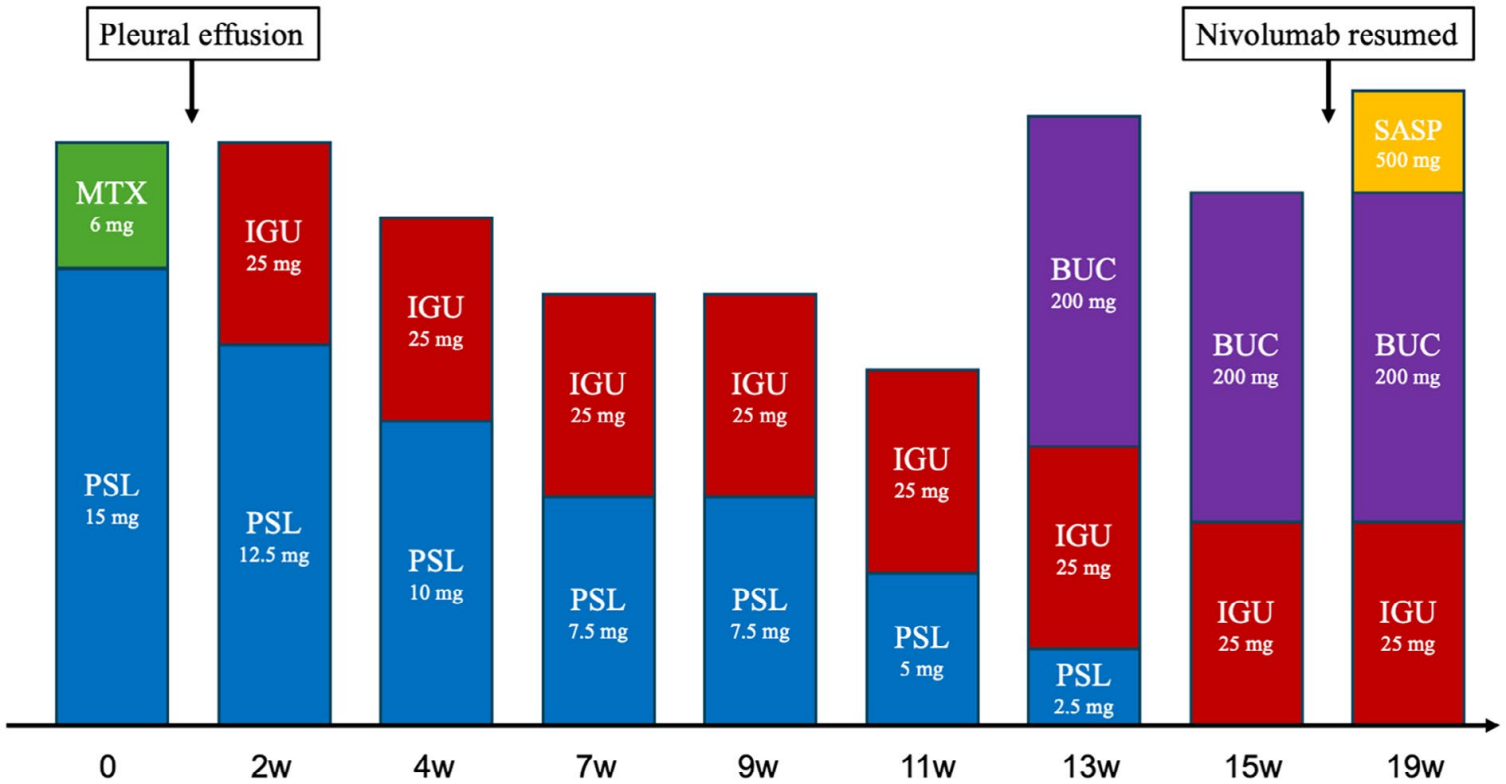
Medications for the treatment of rheumatoid arthritis. Treatment was started with prednisolone (15 mg/day) and methotrexate (6 mg/week). The arrow indicates when the pleural effusion was detected; methotrexate was discontinued after two doses and replaced with iguratimod (25 mg/day). Prednisolone was tapered from 15 mg/day to 2.5 mg/day over 13 weeks and discontinued at 15 weeks. Bucillamine (200 mg/day) was added at 13 weeks. Nivolumab was resumed at 17 weeks, and salazosulfapyridine (500 mg/day) was added at 19 weeks because of mild arthralgia in the left wrist. The rheumatoid arthritis was subsequently controlled with iguratimod, bucillamine, and salazosulfapyridine. BUC, Bucillamine; IGU, Iguratimod; MTX, Methotrexate; PSL, Prednisolone; SASP, Salazosulfapyridine.

## 4 Conclusion and Results

Before nivolumab initiation, the subcutaneous nodule under the umbilical port scar had enlarged to 18.0 mm. After five courses of nivolumab, the nodule decreased to 11.1 mm (a 38% reduction), and this reduction was maintained on the subsequent CT performed 24 weeks later (11.4 mm; Figure 2C), corresponding to a partial response according to RECIST version 1.1. The peritoneal nodules also appeared slightly smaller, but they were less than 10 mm in diameter and were therefore regarded as non‐measurable lesions. No new lesions appeared during nivolumab therapy.

After discontinuation of nivolumab, the nodule remained stable in size during immunosuppressive therapy, whereas CEA levels increased from 11.1 to 34.0 ng/mL (Figure 3). At that time, the RA was well controlled with iguratimod and bucillamine, and nivolumab was therefore resumed. Four weeks after resumption, mild arthralgia in the left wrist developed, which was managed by adding salazosulfapyridine (500 mg/day) without discontinuing nivolumab. A total of 15 courses of nivolumab were administered. Nivolumab was subsequently discontinued because of invasive pulmonary aspergillosis. Although the aspergillosis was treated successfully, the gastric cancer progressed, and the patient died of the primary disease 8 months after the resumption of nivolumab. The total follow‐up period was approximately 16 months from the initiation of nivolumab.

## 5 Discussion

Immune checkpoint inhibitors (ICI) are frequently associated with specific adverse events known as irAEs. Buder‐Bakhaya et al. reported that about 5.1% (10 of 195) of patients treated with a PD‐1 inhibitor with or without ipilimumab developed rheumatic irAEs, which are phenotypically similar to classical rheumatic diseases and include inflammatory arthritis, myositis, vasculitis, and scleroderma [12]. While the incidence of rheumatic irAEs is less well established compared to other irAEs, arthritis has been reported in fewer than 3.0% of patients receiving PD‐1 inhibitor therapy [13]. Among these, cases meeting the classification criteria for rheumatoid arthritis (RA) remain extremely rare. We identified and summarized seven such cases (Table 2) [14, 15]. In addition, nivolumab has also been associated with Sjögren's syndrome as an irAE [16]. Although Sjögren's syndrome is associated with approximately 30% of RA cases [17], to our knowledge, the concurrent development of both conditions associated with nivolumab has not been reported. Therefore, our case may represent a rare but clinically important manifestation of PD‐1 inhibitor‐associated autoimmunity. Several limitations should be noted. Because autoimmune serologies, including RF, anti‐CCP, anti‐SS‐A and anti‐SS‐B antibodies, and ANA, were not evaluated before the initiation of nivolumab, the presence of pre‐existing subclinical autoimmunity cannot be excluded. In particular, the markedly high titers of RF and anti‐CCP antibodies observed at diagnosis may suggest pre‐existing preclinical RA that became clinically evident after PD‐1 blockade. For this reason, we describe these conditions as nivolumab‐associated rather than nivolumab‐induced.

**TABLE 2:** Reported cases of rheumatoid arthritis associated with immune checkpoint inhibitor therapy.

Reported cases of patients with RA after ICI treatment						
Case	Sex	Age	Primary tumor	Time to onset	RA response to treatment	References
1	Female	55	Squamous cell carcinoma of the vagina	< 1 month	Resolution with NSAIDs	[14]
2	Female	66	Endometrial adenocarcinoma	1 month	Resolution with prednisone 10 mg/day	[14]
3	Male	59	Lung adenocarcinoma	1 month	Resolution with prednisone 10 mg/day	[14]
4	Female	56	Metastatic melanoma	1 month	NSAIDs and HCQ 400 mg/day: good response	[14]
5	Male	80	Metastatic melanoma	1 month	Prednisone 15 mg/day and HCQ 200 mg/day: good response	[14]
6	Male	68	Lung adenocarcinoma	1 month	NSAIDs: no effect Stopping nivolumab and MTX 10 mg/week: good response	[14]
7	Male	70	Thoracic esophageal cancer	< 1 month	Stopping nivolumab, SASP 500 → 1000 mg/day and PSL 5 mg/day: good response	[15]

*Note:* Seven previously reported cases fulfilling the classification criteria for rheumatoid arthritis after immune checkpoint inhibitor therapy are summarized. In all cases, the arthritis developed within 1 month after the initiation of therapy and responded to NSAIDs, corticosteroids, or conventional synthetic disease‐modifying antirheumatic drugs.

Abbreviations: HCQ, hydroxychloroquine; ICI, immune checkpoint inhibitor; MTX, methotrexate; NSAIDs, non‐steroidal anti‐inflammatory drugs; PSL, prednisolone; RA, rheumatoid arthritis; SASP, salazosulfapyridine.

Although irAE‐related arthritis and RA are different diseases, there are some similarities. Both irAE‐related arthritis and RA affect the proximal interphalangeal, wrist and knee joints and can cause erosive joint destruction. While irAE‐related arthritis is RF and CCP‐Ab negative and tendon involvement is prominent early in the disease, RA is RF and CCP‐Ab positive and more common in females [18]. In this case, RF and CCP‐Ab were positive; 406 U/mL and 500 U/mL respectively. Symptom onset of irAE‐related arthritis is generally considered to be 100 days after the first administration of PD‐1 inhibitors [12]; however, Belkhir et al. reported that RA developed within 1 month after PD‐1 inhibitor treatment [14]. In this case, symptoms also appeared within 1 month of nivolumab administration. This suggests that RA may have an earlier onset compared to irAE arthritis.

CCP‐Ab targets citrullinated proteins, which are expressed on the surface of the inflamed synovium. CCP‐Ab is not only used to diagnose RA but to predict those who may develop RA. CCP‐Ab is known to be positive in approximately 33.7% of RA patients before the onset of symptoms. Detecting asymptomatic CCP‐Ab positive patients could prevent nivolumab‐associated RA [19]. However, the prevalence of RA is approximately 1%, and the sensitivity and specificity of CCP‐Ab are 50% and 90%, respectively [20, 21]. Therefore, the positive predictive value is only 4.8%, based on Bayes' theorem, meaning CCP‐Ab screening is impractical.

The use of PD‐1 inhibitors can affect the autoimmune response and trigger autoimmune diseases such as RA. Peresolimab has been developed on the basis that dysregulation of the PD‐1/PD‐L1 pathway is implicated in RA and has shown efficacy in the treatment [22]. In RA patients, the expression of PD‐1 on helper T cells in the synovium is elevated and is also associated with disease activity [23]. In addition, the gene expression profile induced by nivolumab treatment is similar to that of RA patients [24]. These findings also suggest that abnormalities in the PD‐1/PD‐L1 pathway may be involved in the pathogenesis of RA [25]. Similarly, in Sjögren's syndrome, it has been reported that PD‐1 is expressed on infiltrating T lymphocytes and PD‐L1 on epithelial cells in the salivary glands, indicating that dysfunction of this PD‐1/PD‐L1 pathway is involved in the breakdown of immune tolerance and the pathogenesis of the disease [26]. Although the precise mechanisms underlying the simultaneous development of RA and Sjögren's syndrome remain unclear, secondary Sjögren's syndrome typically occurs after a long‐standing history of RA in classical autoimmune conditions [17]. In contrast, the abrupt and concurrent onset observed in our case suggests an ICI‐related pathophysiology. Therefore, dysregulation of this pathway induced by ICIs may contribute to the concurrent development of both conditions.

According to the ASCO [9] guidelines for the management of irAE‐related arthritis, mild symptoms with erythema, inflammation, or joint swelling should be treated with acetaminophen or NSAIDs. For moderate symptoms that limit instrumental activities of daily living (ADL), the initiation of systemic corticosteroids (e.g., prednisone) and disease‐modifying antirheumatic drugs (DMARDs) is recommended, and temporary withholding of the ICI should be considered. In cases of severe, disabling symptoms that limit self‐care ADL or cause irreversible joint damage, the ICI must be withheld, and prompt consultation with a rheumatologist is strongly advised. Furthermore, when the condition fulfills the classification criteria for true RA, management should strictly align with standard RA treatment protocols, requiring targeted immunosuppressive therapy with specific DMARDs in close collaboration with a rheumatologist. In contrast, the management of ICI‐induced sicca syndrome primarily focuses on local symptomatic relief. ASCO guidelines recommend the use of artificial tears for dry eyes, and saliva substitutes or secretagogues for dry mouth. Systemic corticosteroids are rarely indicated for sicca syndrome alone, and ICI therapy can often be continued. Even when the condition fulfills the classification criteria for true Sjögren's syndrome, local symptomatic relief remains the standard of care, rather than systemic immunosuppression, in the absence of severe systemic or extraglandular manifestations [27]. When both conditions occur simultaneously, as in the present case, systemic immunosuppressive therapy should prioritize RA, the more aggressive and structurally damaging condition, while symptomatic and supportive care should be concurrently maintained for Sjögren's syndrome.

Generally, the onset of irAEs is associated with a favorable anti‐tumor effect [28]. However, there is a clinical concern that immune‐modulating agents used for severe irAEs might negatively affect tumor response to ICI therapy. Although this remains controversial, Petrelli et al. reported that the systemic administration of low‐ to moderate‐dose corticosteroids to treat irAEs does not adversely affect overall survival and time to treatment failure in patients with melanoma or non‐small cell lung cancer [29]. Regarding DMARDs, conventional synthetic DMARDs do not affect anti‐tumor efficacy, whereas biologic DMARDs can negatively impact tumor response [30].

In the present case, nivolumab achieved a partial response but was discontinued after the ninth course because of worsening arthritis, and moderate‐dose corticosteroids were administered and gradually tapered in combination with conventional synthetic DMARDs (Figure 4). The tumor remained stable in size during this period, and nivolumab was resumed once the arthritis was controlled with conventional synthetic DMARDs alone, without systemic corticosteroids. Although mild arthralgia recurred after resumption, it was managed by adding another conventional synthetic DMARD, and nivolumab could be continued thereafter. However, CEA levels increased during this period, and it therefore cannot be determined from this single case whether the immunosuppressive therapy affected the antitumor efficacy of nivolumab.

We experienced one case in which rheumatoid arthritis and Sjögren's syndrome became evident with nivolumab for recurrent GC. If arthritic symptoms appear early after PD‐1 inhibitor use or symptoms worsen despite NSAID treatment, RA as well as irAE arthritis should be suspected.

## Author Contributions

**Haruhiko Takemoto:** writing – original draft. **Keiji Nishibeppu:** writing – review and editing. **Takeshi Kubota:** writing – review and editing. **Hiroyuki Inoue:** validation. **Kazuya Takabatake:** validation. **Hirotaka Konishi:** validation. **Hitoshi Fujiwara:** validation. **Atsushi Shiozaki:** supervision.

## Funding

The authors have nothing to report.

## Ethics Statement

Ethics review is not required for the publication of case reports at our institution.

## Consent

Written informed consent was obtained from the patient for the publication of this case report and for the use of their clinical information, in accordance with the journal's policy on patient consent.

## Conflicts of Interest

The authors declare no conflicts of interest.

## Data Availability

The datasets used or analyzed in the current study are available from the corresponding author on reasonable request.
